# Selective Hepatic Vascular Exclusion versus Pringle Maneuver in Major Hepatectomy: A Systematic Review and Meta-Analysis

**DOI:** 10.3389/fsurg.2022.860721

**Published:** 2022-04-06

**Authors:** Shahd Mobarak, Martyn C. Stott, Munir Tarazi, Rebecca J. Varley, Madhav S. Davé, Minas Baltatzis, Thomas Satyadas

**Affiliations:** ^1^ Department of Hepato-Pancreato-Biliary Surgery, Manchester Royal Infirmary, Manchester, UK; ^2^ Department of Surgery and Cancer, Imperial College London, London, UK; ^3^ Department of Upper GI Surgery, Salford Royal Hospital, Salford, UK

**Keywords:** pringle, selective hepatic vascular exclusion (SHVE), hepatectomy, liver resection, Systematic (Literature) Review

## Abstract

**Objectives:**

Mortality and morbidity following hepatic resection is significantly affected by major intra-operative blood loss. This systematic review and meta-analysis evaluates whether selective hepatic vascular exclusion (SHVE) compared to a Pringle maneuver in hepatic resection reduces rates of morbidity and mortality.

**Methods:**

A systematic review and meta-analysis were conducted according to the PRISMA guidelines by screening EMBASE, MEDLINE/PubMed, CENTRAL and SCOPUS for comparative studies meeting the inclusion criteria. Pooled odds ratios or mean differences were calculated for outcomes using either fixed- or random-effects models.

**Results:**

Six studies were identified: three randomised controlled trials and three observational studies reporting a total of 2,238 patients. Data synthesis showed significantly decreased rates of mortality, overall complications, blood loss, transfusion requirements, air embolism, liver failure and multi-organ failure in the SHVE group. Rates of hepatic vein rupture, post-operative hemorrhage, operative and warm ischemia time, length of stay in hospital and intensive care unit were not statistically significant between the two groups.

**Conclusion:**

Performing SHVE in major hepatectomy may result in reduced rates of morbidity and mortality when compared to a Pringle maneuver. The results of this meta-analysis are based on studies where tumors were adjacent to major vessels. Further RCTs are required to validate these results.

**Clinical Trial Registration:**

PROSPERO (CRD42020212372) https://www.crd.york.ac.uk/prospero/display_record.php?RecordID=212372.

## Introduction

Over the past two decades, improvements in safety have allowed hepatic resection to play a significant role in the management of benign and malignant hepatobiliary disease (1–4). Due to the liver’s specialized blood supply, major intra-operative hemorrhage can significantly affect morbidity and mortality (5, 6). Most hepatic resections require vascular occlusion, especially where tumors are sizeable or lie close to major vessels.

The Pringle maneuver, first described in 1908 as a technique to minimize blood loss during hepatic surgery, is the most common technique of vascular occlusion in surgical practice (7–9). It involves clamping of the hepatoduodenal ligament and occluding the portal triad, which minimizes the blood inflow into the liver via the portal vein and hepatic artery. Blood outflow from the liver is not affected, therefore Pringle maneuver cannot prevent backflow bleeding from the hepatic veins. Furthermore, if the tumor lies close to the inferior vena cava or at the confluence of one or more of the major hepatic veins, major hemorrhage as well as air embolism can occur, as a result of injury of these vessels. Total hepatic vascular exclusion (THVE) was developed in an attempt to reduce these complications, occluding both hepatic inflow and outflow by performing a Pringle maneuver and clamping the inferior vena cava (IVC) above and below the liver (10–12). However, this causes significant hemodynamic disturbance due to the interruption of venous blood flow in the IVC (13, 14).

Selective hepatic vascular exclusion (SHVE) is a newer technique which involves clamping the hepatic veins without clamping the IVC (15, 16). This can control hepatic inflow and outflow, preserving caval flow and therefore avoiding major hemodynamic disturbance. SHVE is not widely used by surgeons despite the theoretical advantage it offers, as it is technically challenging and can be complicated by laceration of the hepatic veins during dissection resulting in major hemorrhage.

The safest type of vascular occlusion to perform in hepatectomy remains a contested topic of discussion. The aim of this systematic review and meta-analysis is to compare morbidity and mortality between SHVE and Pringle maneuver in major hepatectomy surgery.

## Methods

### Study Design

This systematic review and meta-analysis was registered at PROSPERO (CRD42020212372). It was conducted according to the Preferred Reporting Items for Systematic Reviews and Meta-Analyses (PRISMA).

### Data Sources and Search Strategy

The following electronic databases were searched: MEDLINE/PubMed (1946 to June 2021), EMBASE (1947 to June 2021), Scopus and the Cochrane Central Register of Controlled Trials (CENTRAL) from The Cochrane Library (2020, Issue 7) on 26 June 2021. This was done by two independent authors (SM, MT). A combination of medical subject headings (MeSH) and free-text terms were used to form the search strategy for each database. This is displayed in Supplementary Table S1 (Online Resource 1).

In order to identify relevant studies that did not get included in the initial database searches, the reference lists of selected articles were examined. The World Health Organization International Clinical Trials Registry, ClinicalTrials.gov, ISRCTN Register and PROSPERO were also searched to identify any unpublished studies.

### Study Selection

Our inclusion criteria included: randomized controlled trials (RCTs) or comparative observational studies in the English language; human studies; studies including patients aged 18 years or older of any gender; studies where a hepatectomy was performed; studies where a Pringle maneuver was performed for hepatic inflow occlusion in the SHVE group.

Our exclusion criteria included: non-English studies; non-medical, non-human studies; studies in patients under the age of 18 years old and conference abstracts, editorials, expert opinion, case reports and non-comparative observational studies.

The studies that were identified by the initial search strategy were reviewed by two independent authors (RV, MT). Duplicated were removed. Rayyan software was used to screen titles and abstracts of identified studies for inclusion into the review (17). If the study abstract was not sufficient to make a decision for inclusion, the full paper was screened. Any conflicts that arose were resolved through discussion, and a third author (MD) made the final decision where necessary.

### Data Extraction

The data was extracted from studies using an electronic data extraction spreadsheet. This was done by two independent authors (SM, MCS). Any conflicts that arose were resolved through discussion, and a third author (MD) made the final decision where necessary. Collected data included: study-related data, patient demographics, peri-operative management and relevant outcome measures.

### Outcome Measures

Intra-operative outcome measures included: operative time (minutes), warm ischemia time (minutes), blood loss (milliliters), patients requiring blood transfusion, blood transfusion (units), air embolism and hepatic vein rupture.

Post-operative outcome measures included: overall mortality, intra-operative mortality, in-hospital mortality, overall complication rate (%), hospital stay (days), intensive care unit (ICU) stay (days), post-operative hemorrhage, liver failure and multi-organ failure.

Where studies reported outcomes as median with range, the mean and standard deviation were estimated using the validated method described by Hozo et al. (18).

### Assessment of Risk of Bias

Risk of bias was assessed by two independent authors (SM, MCS). This was carried out using the revised Cochrane risk-of-bias tool (RoB 2) for RCTs and the Cochrane Risk Of Bias In Non-Randomized Studies – of Interventions tool (ROBINS-I) for non-randomized studies. Where there were disagreements between the two authors, this was discussed and the final decision was made by a third independent author (MD).

### Data Synthesis and Statistical Analysis

The software Review Manager (RevMan) (The Cochrane Collaboration; Version 5.3.5, The Nordic Cochrane Centre, Copenhagen, Denmark) was used for data synthesis (19). This was done by one independent author (MB) who entered the extracted data into the software. A second independent author (MD) then reviewed the entered data.

To estimate treatment effects, relevant outcome parameters that were extracted from the included studies were assessed. For dichotomous variables, the Mantel-Haenszel method was used to pool the odds ratio (OR). For continuous variables, the mean difference (MD) was calculated between the two groups (20). A forest plot was generated for each outcome measure with 95% confidence intervals (CIs) and its associated p-value. Statistical significance was defined as *p* < 0.05.

The Cochran Q test (χ^2^) was used to assess the heterogeneity between studies. This was then further quantified by generating an inconsistency statistic (*I*^2^) for each outcome measure. Low heterogeneity was defined as an *I*^2^ of 0–50% and fixed-effects modelling was used. Conversely, high heterogeneity was defined as an *I*^2^ of 51–100% and random-effects modelling was used.

To explore potential sources of heterogeneity, sensitivity analyses were carried out. For each outcome parameter with high inter-study heterogeneity, individual studies were removed and the analysis would be repeated to assess that study’s contribution to the overall effect size and heterogeneity. In order to explore potential changes in the effect size, subgroup analyses of the RCTs and observational studies were carried out.

The independent (unpaired) samples t-test was performed on the Pringle and SHVE groups to assess statistical significance between patient demographics. This was done using the software IBM SPSS Statistics (IBM Corp; Version 23.0, Armonk, NY, USA) (21). Statistical significance was defined as *p* < 0.05.

## Results

### Study Selection

**Figure 1:**
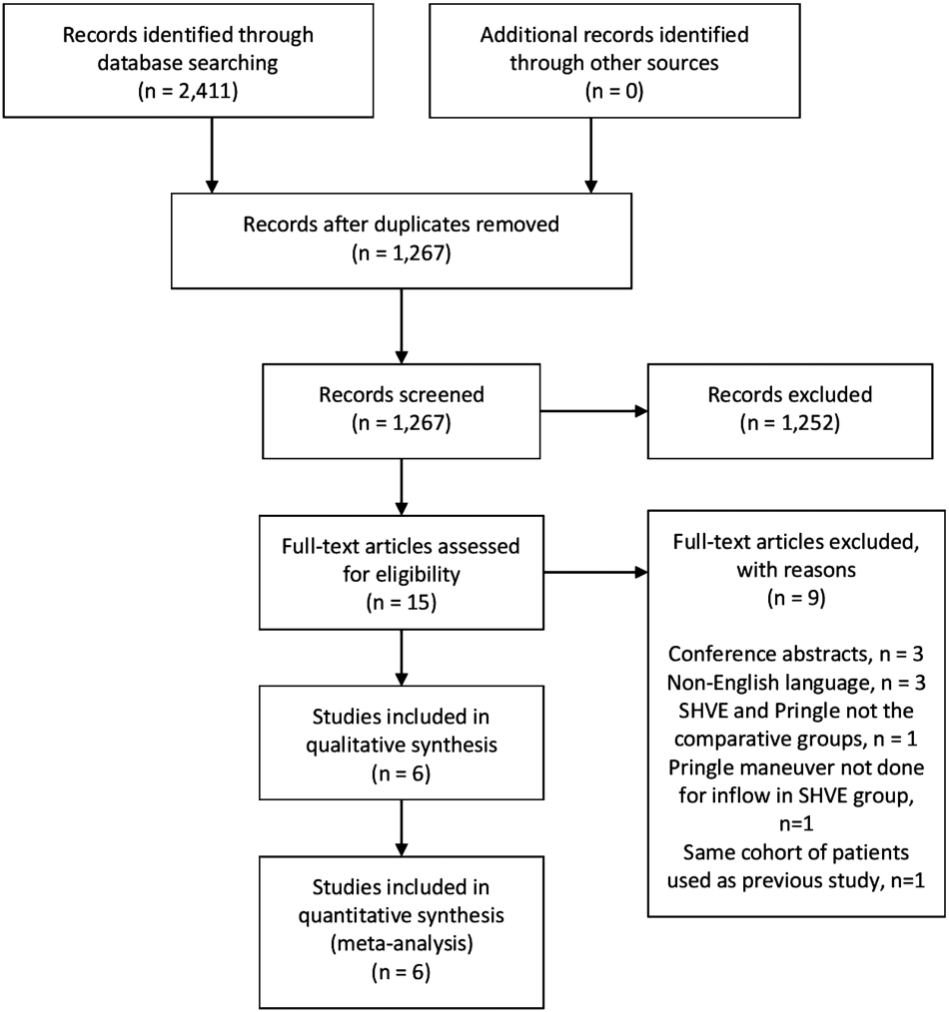
PRISMA flowchart.

The literature search identified 2,411 studies, which became 1,267 following removal of duplicated studies. Abstracts were then assessed for eligibility and 1,253 studies were excluded. From the remaining 15 studies, six met the inclusion criteria. Therefore the study population for this systematic review is comprised of three RCTs, two retrospective cohort studies and one case-control study reporting a total of 2,238 patients. PRISMA flowchart is demonstrated in Figure 1.

### Study Characteristics

All studies were published between the years 2003 and 2019. One study was undertaken in Greece (22), four in China (23–26) and one in Thailand (27). All RCTs were single-center studies. Study durations ranged from 11 to 96 months. In total, there were 1,288 patients in the Pringle group and 950 in the SHVE group. Study characteristics are presented in Table 1.

**Table 1:** Summary of characteristics of included studies.

Author	Year	Journal	Country	Study design	Retrospective or prospective	Study period	Study duration (months)	*n*, total	*n*, Pringle	*n*, SHVE
Smyrniotis et al.	2003	World Journal of Surgery	Greece	RCT	Prospective	1995–2002	84	110	55	55
Zhou et al.	2008	European Journal of Surgical Oncology	China	Case-control	Retrospective	2000–2005	58	235	110	125
Zhang et al.	2012	British Journal of Surgery	China	Cohort	Retrospective	2003–2010	84	1420	870	550
Yang et al.	2014	American Surgeon	China	Cohort	Retrospective	2003–2011	96	273	153	120
Si-Yuan et al.	2014	International Journal of Surgery	China	RCT	Prospective	2008–2010	24	160	80	80
Tongsiri et al.	2020	Journal of The Medical Association of Thailand	Thailand	RCT	Prospective	2018–2019	11	40	20	20

*RCT, randomized controlled trial; SHVE, selective hepatic vascular exclusion.*

Baseline demographics of the study populations are presented in Table 2. There was no statistically significant difference in the mean age and gender between the Pringle and SHVE groups. Tumor size, number of patients with cirrhosis (including Child-Pugh Grade) and hepatitis B status were only reported in a few of the studies, but where they were reported, they were comparable across the two groups. The extent of tumor invasion of the hepatic veins was reported by the four studies from China and remained comparable across the two groups (23–26). These studies only selected patients with tumors encroaching on the hepatic veins. Zhou et al. (23) and Tongsiri et al. (27) reported the number of hepatic veins involved rather than named veins therefore it was not possible to assess whether there were differences between the Pringle and SHVE groups.

**Table 2:** Baseline patient demographics of included studies.

Author	Average age (years)		*n*, male		Tumour size (cm)		*n*, cirrhosis		*n*, Child-Pugh Grade A		*n*, Child-Pugh Grade B		*n*, HBsAg + ve	
	Pringle	SHVE	Pringle	SHVE	Pringle	SHVE	Pringle	SHVE	Pringle	SHVE	Pringle	SHVE	Pringle	SHVE
Smyrniotis et al.	62	61	44	43	N/R	N/R	N/R	N/R	N/R	N/R	N/R	N/R	N/R	N/R
Zhou et al.	52.3	51.6	77	86	11.8	12.4	65	74	102	113	8	12	71	90
Zhang et al.	53	51	630	406	8.6	8.9	604	393	580	379	24	14	621	427
Yang et al.	41.9	45.8	62	41	12.9	14.2	2	1	N/R	N/R	N/R	N/R	N/R	N/R
Si-Yuan et al.	48.3	49.2	63	61	8	8.2	48	50	43	45	5	5	N/R	N/R
Tongsiri et al.	57.4	61.1	4	11	N/R	N/R	N/R	N/R	N/R	N/R	N/R	N/R	1	0
Independent samples t-test	*p* = 0.840		*p* = 0.743		*p* = 0.758		*p* = 0.776		*p* = 0.771		*p* = 0.780		*p* = 0.815	

*Statistical significance defined as p* *< 0.05.*

*HBsAg, hepatitis B surface antigen; N/R, not reported.*

Other than one study that solely looked at outcomes in hemangioma (25), malignancy was the most common indication for resection, and hepatocellular carcinoma (HCC) accounted for the majority of malignant lesions in both groups. The number of patients with HCC were similar across the two groups. The most commonly performed resections were right and left hepatectomy, with very few numbers reported for the various segmentectomies, and this remained comparable across the two groups. All studies reported the use of a clamp-crushing technique for liver resection, except Tongsiri et al. which used ultrasonic dissection (27), and all studies used additional polypropylene 3-0 and 4-0 sutures for hemostasis.

Two studies performed a continuous Pringle maneuver for all patients in both groups (22–25) and Tongsiri et al. performed intermittent Pringle maneuver for both groups (27). Si-Yuan et al. performed a continuous Pringle maneuver for the SHVE group only (26) and two studies performed a continuous Pringle maneuver either if the liver was cirrhotic (24) or developed cirrhosis (23) in both groups. Zhang et al. (24) and Yang et al. (25) converted to THVE in the Pringle group in 34 and 11 patients respectively. Si-Yuan et al. converted to THVE in one patient in the SHVE group as the tumor had invaded the IVC (26). Tongsiri et al. converted two patients in the SHVE group to Pringle (27). All studies described clamping the right, middle and left hepatic veins in all cases regardless of the type of resection. Operative techniques are presented in Supplementary Table S2 (Online Resource 1).

### Data Synthesis

#### Hemorrhage and Transfusion

The pooled analysis demonstrated a statistically significant decrease in blood loss (MD: −353.13, 95% CI: −380.80– −325.46, *p* < 0.00001); number of patients requiring blood transfusion (OR: 0.31, 95% CI: 0.20–0.50, *p* < 0.00001); and number of units transfused (MD: −1.59, 95% CI: −1.70– −1.49, *p* < 0.00001) in the SHVE group compared to the Pringle group. Forest plots for these outcomes are presented in Table 3. Rates of post-operative hemorrhage remained similar between the two groups (OR: 0.55, 95% CI: 0.17–1.78, *p* = 0.32). This is presented in Supplementary Figure S3 (Online Resource 1). Heterogeneity between studies for blood loss (*I*^2^ = 0%, *p* = 0.72); units of blood transfused (*I*^2^ = 0%, *p* = 0.48); and rates of post-operative hemorrhage (*I*^2 ^= 22%, *p* = 0.27) was low. There was high heterogeneity between studies for number of patients requiring blood transfusion (*I*^2^ = 74%, *p* = 0.004).

**Table 3:** Forest plots comparing primary and secondary outcomes in Pringle and selective hepatic vascular exclusion.

Blood loss	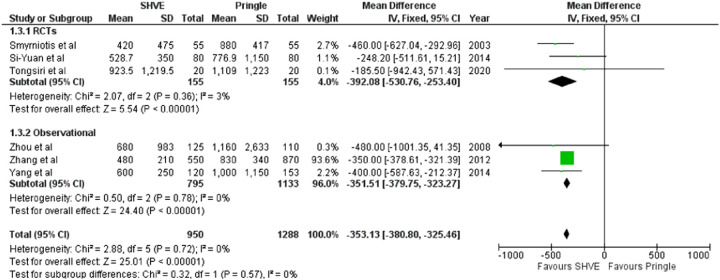
Patients requiring transfusion	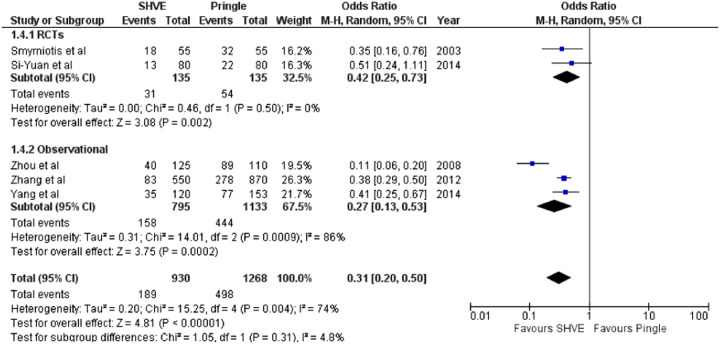
Units of blood transfused	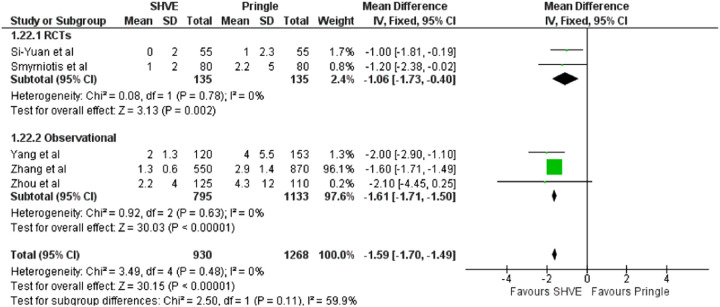
Overall mortality	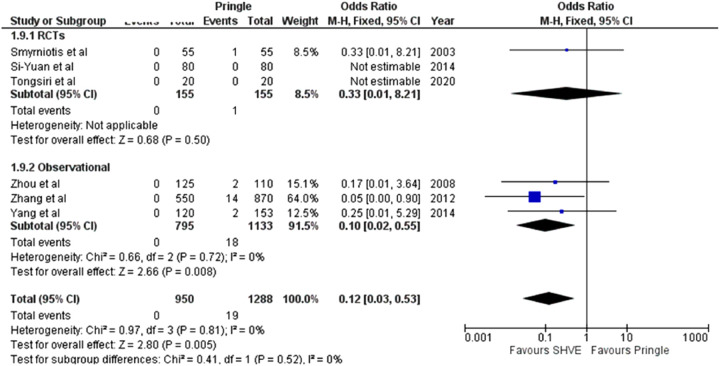
Overall complications	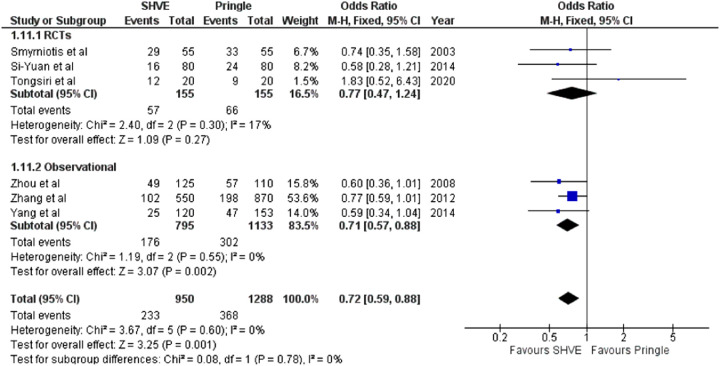
Hepatic vein rupture	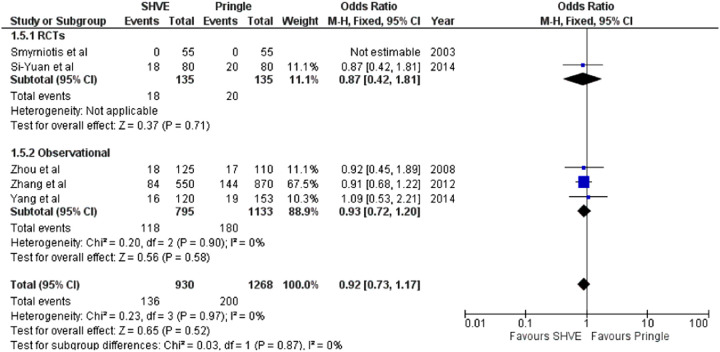
Bile leak	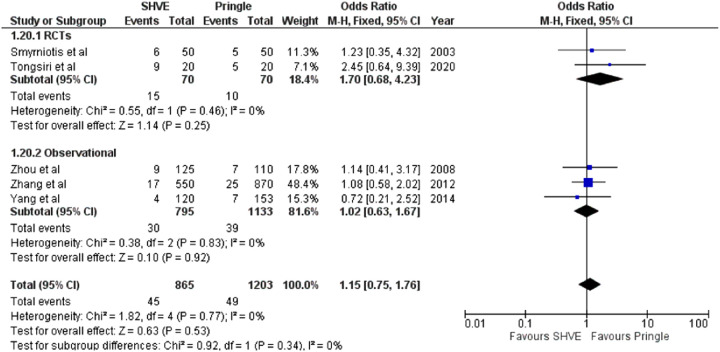
Air embolism	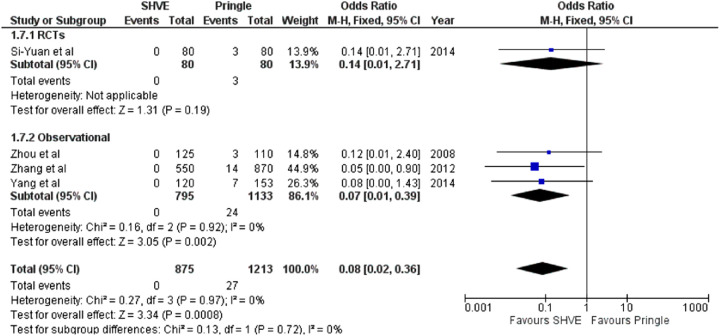
Liver failure	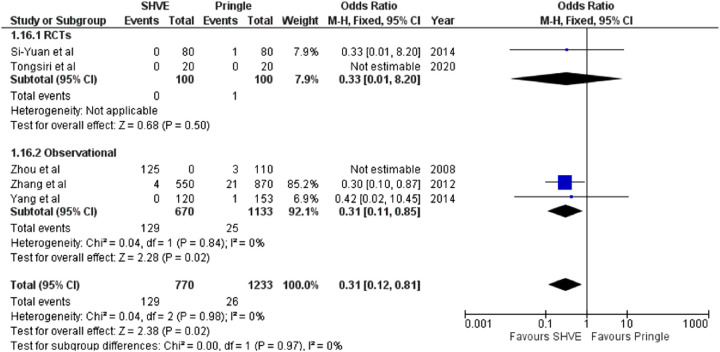
Multi-organ failure	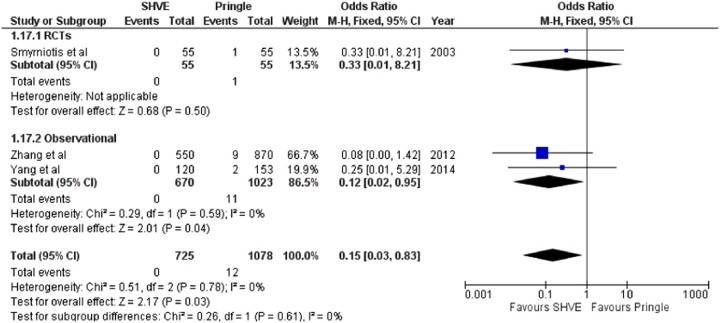

*CI, confidence interval; M-H, Mantel-Haenszel test; RCT, randomized controlled trial.*

#### Morbidity and Mortality

There was a statistically significant decrease in overall mortality (OR: 0.12, 95% CI: 0.03–0.55, *p* = 0.005) and in-hospital mortality (OR: 0.09, 95% CI: 0.01–0.68, *p* = 0.02) in the SHVE group compared to the Pringle group. Heterogeneity remained low between the studies for both overall mortality (*I*^2^ = 0%, *p* = 0.81) and in-hospital mortality. (*I*^2^ = 0%, *p* = 0.41). The forest plot for overall mortality is presented in Table 3. In-hospital mortality is presented in a forest plot in Supplementary Figure S4 (Online Resource 1).

The meta-analysis of complication rate demonstrated a statistically significant decrease in the SHVE group compared to the Pringle group (OR: 0.72, 95% CI: 0.59–0.88, *p* = 0.001) with low heterogeneity (*I*^2^ = 0%, *p* = 0.60). There was no statistically significant difference in hepatic vein rupture (OR: 0.92, 95% CI: 0.73–1.17, *p* = 0.52) or bile leak (OR: 1.15, 95% CI: 0.75–1.76, *p* = 0.53) between the two groups. Heterogeneity was low for both hepatic vein rupture (*I*^2^ = 0%, *p* = 0.97) and bile leak (*I*^2 ^= 0%, *p* = 0.77). There was a statistically significant decrease in air embolism (OR: 0.08, 95% CI: 0.02–0.36, *p* = 0.0008); liver failure (OR: 0.31, 95% CI: 0.12–0.81, *p* = 0.02); and multi-organ failure (OR: 0.15, 95% CI: 0.03–0.83, *p* = 0.03) in the SHVE group compared to the Pringle group. Heterogeneity between studies for air embolism (*I*^2^ = 0%, *p* = 0.97); liver failure (*I*^2^ = 0%, *p* = 0.98); and multi-organ failure (*I*^2^ = 0%, *p* = 0.78) remained low. The forest plots for these outcomes are presented in Table 3.

#### Duration of Surgery and Hospital Stay

There was no statistically significant difference in warm ischemia (MD: −0.84, 95% CI: −2.18–0.51, *p* = 0.22) or operative time (MD: 6.44, 95% CI: −2.65–15.54, *p* = 0.16) in the SHVE group compared to the Pringle group. Heterogeneity was high for both warm ischemia time (*I*^2^ = 69%, *p* = 0.01) and operative time (*I*^2^ = 84%, *p* < 0.00001).

Similarly, there was no statistically significant difference in length of stay in hospital (MD: −3.04, 95% CI: −8.06–1.98, *p* = 0.24) or ICU (MD: 0.66, 95% CI: −0.53–1.86, *p* = 0.28) in the SHVE group compared to the Pringle group. Heterogeneity was high for both hospital stay (*I*^2^ = 99%, *p* < 0.00001) and ICU stay (*I*^2^ = 99%, *p* < 0.00001). The forest plots for these outcomes are presented in Supplementary Figures S5–S8 (Online Resource 1).

### Sensitivity and Subgroup Analysis

Random-effects modelling was applied to patients requiring transfusion, operative time, warm ischemia time, ICU stay and hospital stay due to the high heterogeneity between studies. This did not affect the pooled effect size or heterogeneity. Sensitivity analyses were also performed. Excluding the Si-Yuan study resulted in the operative time becoming significantly shorter in the Pringle group, excluding the Si-Yuan study resulted in warm ischemia time became significantly shorter in the SHVE group and excluding the Zhang study resulted in the hospital stay becoming significantly shorter in the SHVE group.

Subgroup analyses separating RCTs from observational studies had no effect on the meta-analysis of all outcomes, except complication rate which did not show a significant difference between the SHVE and Pringle groups in RCTs alone and hospital stay which became significantly reduced in the SHVE group.

### Methodological Quality of Included Studies

Overall, risk of bias was low for all randomized controlled trials included in this review. Double blinding was not possible as surgeons knew whether they were performing performed SHVE or Pringle maneuver. Since knowledge of assigned intervention did not affect objectively measured post-operative endpoints, this did not add risk of observer bias. Additionally, there was no bias from missing outcome data and any deviations from intended interventions were equally distributed between both groups. Measurement of outcomes and reporting of results did not confer a significant risk of bias.

Overall, risk of bias was moderate for all observational studies. This was mainly due to issues with confounding bias as studies did not account for important variables or make reasonable adjustments to prevent this. All studies were found to have moderate risk of bias in the selection of reported results. Zhang et al. and Yang et al. had serious issues with deviation from intended interventions. The risk of bias assessment of both RCTs and observational studies is presented in Supplementary Figure 9 (Online Resource 1).

## Discussion

Mortality following major hepatic resection has markedly improved in recent years due to advancements in surgical and anesthetic techniques (1–4). Resection of tumors lying adjacent to the hepatic veins can result in major hemorrhage or venous air embolism. Therefore, hepatic vascular control has been recognized as an important aspect of reducing morbidity in these patients. Whilst portal triad clamping (Pringle maneuver) can control hepatic inflow, it does not prevent backflow from the hepatic veins. THVE may prevent massive bleeding from lacerated veins but causes significant hemodynamic disturbance due to obstruction of blood returning via the IVC. SHVE combines the advantages of both the Pringle and THVE techniques, reducing blood in the hepatic field whilst maintaining caval flow (7–14).

This systematic review and meta-analysis was conducted to compare the mortality and morbidity when using SHVE versus a Pringle maneuver in hepatectomy. Meta-analysis of the data revealed significantly decreased rates of mortality, overall complications, blood loss, blood transfusion rates, units of blood transfused, air embolism, liver failure and multi-organ failure when performing SHVE compared to a Pringle maneuver. The heterogeneity between studies for all these outcomes except blood transfusion rates were low suggesting that these outcomes are robust and reliable. Rates of hepatic vein rupture, post-operative hemorrhage, operative time, warm ischemia time, hospital stay and ICU stay were not statistically significant between the two techniques. All of these outcomes, except for hepatic vein rupture, had high heterogeneity between studies.

The results of this study are consistent with a meta-analysis reported in 2008 comparing techniques of vascular exclusion with Pringle (28). The study by Smyrniotis et al. (22) reported a subgroup analysis of SHVE versus Pringle and showed a significant decrease in blood loss and patients requiring blood transfusion in the SHVE group. There are no registered ongoing trials comparing SHVE to Pringle. Therefore, this review remains the most up to date review of the evidence.

Three of the studies in this review, including one RCT, selected patients who had tumors lying adjacent to the major hepatic veins (24–26). These studies were included the most frequently in the meta-analyses for all outcomes and therefore it is likely that these results suggest that SHVE may be a more appropriate technique to perform in this population of patients.

### Limitations

This review is limited largely by heterogeneity of included studies. Several selection bias can be identified: status of the liver pre-operatively, number and location of resected liver nodules, continuous versus intermittent Pringle maneuver, transection techniques and peri-operative chemotherapy. As chemotherapy affects the quality of the liver parenchyma and subsequently the blood loss, the lack of this information increases the heterogeneity in the results.

Although SHVE describes the technique of hepatic outflow occlusion, there are different methods in which inflow occlusion can be performed. In this review, studies were only included if they performed a Pringle maneuver as part of the hepatic inflow. This minimized the heterogeneity between the studies, but in doing so, reduced the number of good quality studies that could be included in this review. Further studies with a standardized definition of SHVE are required.

This review demonstrated that rates of hepatic vein injury during both liver parenchymal and hepatic vein dissection remains comparable between Pringle and SHVE techniques. However, the studies included reported three different methods for outflow occlusion (ligation, clamping and tourniquet). Although different outflow occlusion techniques increases heterogeneity amongst the studies, this had no effect on rates of hepatic vein laceration.

SHVE is not widely practiced as it is considered technically challenging owing to the difficulty in isolating the major hepatic veins from the vena cava and the risk of injury associated with it. In clinical practice, SHVE is is much less reproducible than the Pringle maneuver, especially in centers with low volume and experience. SHVE has also become less practiced since the publication of many of these studies, partly due to the advance of the laparoscopic approach. Due to the difficulty in comparing existing variables and the low numbers of studies included in this review, wider conclusions for clinical practice cannot be drawn.

## Conclusion

This systematic review and meta-analysis of best available evidence revealed that performing SHVE in major hepatectomy resulted in a lower overall mortality, lower complication rates including air embolism and liver failure and lower amounts of blood loss and transfusion requirement. The results of this meta-analysis are based on few high-quality studies where tumors were adjacent to major vessels, which seems the most suitable situation to utilize this technique. Due to the limitations of this review, it is difficult to draw conclusions for clinical practice. Larger studies are required to identify which groups of patients, tumors and types of resection benefit the most from the use of SHVE.

## Data Availability

The original contributions presented in the study are included in the article/supplementary material, further inquiries can be directed to the corresponding author/s.

## References

[1] Belghiti J Hiramatsu K Benoist S Massault P Sauvanet A Farges O. Seven hundred forty-seven hepatectomies in the 1990s: an update to evaluate the actual risk of liver resection. J Am Coll Surg. (2000) 191(1):38–46. 10.1016/S1072-7515(00)00261-1 10898182

[2] Jarnagin WR Gonen M Fong Y DeMatteo RP Ben-Porat L Little S Improvement in perioperative outcome after hepatic resection: analysis of 1,803 consecutive cases over the past decade. Ann Surg. (2002) 236(4):397–406. 10.1097/00000658-200210000-00001 12368667 PMC1422593

[3] Imamura H Seyama Y Kokudo N Maema A Sugawara Y Sano K One thousand fifty-six hepatectomies without mortality in 8 years. Arch Surg. (2003) 138(11):1198–206. 10.1001/archsurg.138.11.1198 14609867

[4] Fan ST Mau Lo C Poon RT Yeung C Leung Liu C Yuen WK Continuous improvement of survival outcomes of resection of hepatocellular carcinoma: a 20-year experience. Ann Surg. (2011) 253(4):745–58. 10.1097/SLA.0b013e3182111195 21475015

[5] Shimada M Matsumata T Akazawa K Kamakura T Itasaka H Sugimachi K Estimation of risk of major complications after hepatic resection. Am J Surg. (1994) 167:399–403. 10.1016/0002-9610(94)90124-4 8179084

[6] Wei AC Tung-Ping Poon R Fan ST Wong J. Risk factors for perioperative morbidity and mortality after extended hepatectomy for hepatocellular carcinoma. Br J Surg. (2003) 90(1):33–41. 10.1002/bjs.4018 12520572

[7] Pringle JHV. Notes on the arrest of hepatic hemorrhage due to trauma. Ann Surg. (1908) 48(4):541–9. 10.1097/00000658-190810000-00005 17862242 PMC1406963

[8] Dixon E Vollmer CM Jr Bathe OF Sutherland F. Vascular occlusion to decrease blood loss during hepatic resection. Am J Surg. (2005) 190(1): 75–86. 10.1016/j.amjsurg.2004.10.007 15972177

[9] Belghiti J Noun R Malafosse R Jagot P Sauvanet A Pierangeli F Continuous versus intermittent portal triad clamping for liver resection: a controlled study. Ann Surg. (1999) 229(3):369–75. 10.1097/00000658-199903000-00010 10077049 PMC1191702

[10] Heaney JP Stanton WK Halbert DS Seidel J Vice T. An improved technic for vascular isolation of the liver: experimental study and case reports. Ann Surg. (1989) 163:237–41. 10.1097/00000658-196602000-00013 PMC1477087 4286023

[11] Bismuth H Castaing D Garden OJ. Major hepatic resection under total vascular exclusion. Ann Surg. (1989) 210:13–9. 10.1097/00000658-198907000-00002 2742411 PMC1357759

[12] Berney T Mentha G Morel P. Total vascular exclusion of the liver for the resection of lesions in contact with the vena cava or the hepatic veins. Br J Surg. (1998) 85:485–58. 10.1046/j.1365-2168.1998.00659.x 9607528

[13] Delva E Barberousse JP Nordlinger B Ollivier JM Vacher B Guilmet C Hemodynamic and biochemical monitoring during major liver resection with use of hepatic vascular exclusion. Surgery. (1984) 95(3): 309–18. 6701787

[14] Belghiti J Noun R Zante E Ballet T Sauvanet A. Portal triad clamping or hepatic vascular exclusion for major liver resection. A controlled study. Ann Surg. (1996) 224:155–61. 10.1097/00000658-199608000-00007 8757378 PMC1235336

[15] Elias D Lasser P Debaene B Doidy L Billard V Spencer A Intermittent vascular exclusion of the liver (without vena cava clamping) during major hepatectomy. Br J Surg. (1995) 82(11):1535–9. 10.1002/bjs.1800821126 8535812

[16] Cherqui D Malassagne B Colau PI Brunetti F Rotman N Fagniez PL. Hepatic vascular exclusion with preservation of the caval flow for liver resections. Ann Surg. (1999) 230(1):24–30. 10.1097/00000658-199907000-00004 10400032 PMC1420840

[17] Ouzzani M Hammady H Fedorowicz Z Elmagarmid A. Rayyan – a web and mobile app for systematic reviews. Syst Rev. (2016) 5:210. 10.1186/s13643-016-0384-4 27919275 PMC5139140

[18] Hozo SP Djulbegovic B Hozo I. Estimating the mean and variance from the median, range, and the size of a sample. BMC Med Res Methodol. (2005) 5:13. 10.1186/1471-2288-5-13 15840177 PMC1097734

[19] Review Manager (RevMan) [computer program]. Version 5.4. The Cochrane Collaboration (2020).

[20] Mantel N Haenszel W. Statistical aspects of the analysis of data from retrospective studies of disease. J Natl Cancer Inst. (1959) 22(4):719–48. 13655060

[21] IBM SPSS Statistics for Windows [computer program]. Version 23.0. Armonk, NY (2015).

[22] Smyrniotis VE Kostopanagiotou GG Contis JC Farantos CI Voros DC Kannas DC Selective hepatic vascular exclusion versus Pringle maneuver in major liver resections: prospective study. World J Surg. (2003) 27(7):765–9. 10.1007/s00268-003-6978-8 14509502

[23] Zhou W Li A Pan Z Fu S Yang Y Tang L Selective hepatic vascular exclusion and Pringle maneuver: a comparative study in liver resection. Eur J Surg Oncol. (2008) 34(1):49–54. 10.1016/j.ejso.2007.07.001 17709229

[24] Zhang J Lai EC Zhou WP Fu S Pan Z Yang Y Selective hepatic vascular exclusion versus Pringle maneuver in liver resection for tumors encroaching on major hepatic veins. Br J Surg. (2012) 99(7):973–7. 10.1002/bjs.8764 22539200

[25] Yang Y Zhao LH Fu SY Lau WY Lai EC Gu FM Selective hepatic vascular exclusion versus pringle maneuver in partial hepatectomy for liver hemangioma compressing or involving the major hepatic veins. Am Surg. (2014) 80(3):236–40. 10.1177/000313481408000317 24666863

[26] Si-Yuan F Yee LW Yuan Y Sheng-Xian Y Zheng-Guang W Gang H Pringle maneuver versus selective hepatic vascular exclusion in partial hepatectomy for tumors adjacent to the hepatocaval junction: a randomized comparative study. Int J Surg. (2014) 12(8):768–73. 10.1016/j.ijsu.2014.05.068 24907420

[27] Tongsiri N Siripornadulsilp S Impool T. Comparison of early clinical outcomes between intermittent vascular inflow occlusion versus intermittent selective hepatic vascular exclusion in hepatic resections for cholangiocarcinoma patients: a prospective randomized controlled trial study. J Med Assoc Thai. (2020) 103:521–8. 10.35755/jmedassocthai.2020.06.11023

[28] Rahbari NN Koch M Mehrabi A Weidmann K Motschall E Kahlert C Portal triad clamping versus vascular exclusion for vascular control during hepatic resection: a systematic review and meta-analysis. J Gastrointest Surg. (2009) 13(3):558–68. 10.1007/s11605-008-0588-6 18622655

